# STAT3: An Emerging Therapeutic Target for Hepatocellular Carcinoma

**DOI:** 10.3390/cancers11111646

**Published:** 2019-10-25

**Authors:** Carol Lee, Siu Tim Cheung

**Affiliations:** 1 Department of Surgery, The Chinese University of Hong Kong, Hong Kong, China; carollee@surgery.cuhk.edu.hk; 2 Li Ka Shing Institute of Health Sciences, The Chinese University of Hong Kong, Hong Kong, China

**Keywords:** STAT3, transcription factor, targeted therapy, combination therapy, hepatocellular carcinoma

## Abstract

Hepatocellular carcinoma (HCC) is a major global health problem and its treatment options have been limited. Signal transducer and activator of transcription 3 (STAT3) is a transcription factor important for various cellular processes. Overexpression and constitutive activation of STAT3 have been frequently found in HCC and associated with poor prognosis. Ample evidence has shown that STAT3 plays pivotal roles in the initiation, progression, metastasis and immune suppression of HCC. Thus, STAT3 has attracted attention as a novel therapeutic target in HCC. Clinical trials have investigated STAT3-targeted therapeutics either as monotherapy or in combination with chemotherapeutic agents, immune checkpoint inhibitors and alternative targeted drugs. Some of these studies have yielded encouraging results. Particularly, napabucasin—a cancer stemness inhibitor targeting STAT3-driven gene transcription—has stood out with its promising clinical efficacy and safety profile. Nonetheless, clinical investigations of STAT3-targeted therapies in HCC are limited and more efforts are strongly urged to evaluate their clinical performance in HCC. Here, we provide a comprehensive review of the roles of STAT3 in HCC and follow by comprehensive analysis of STAT3 targeted strategies.

## 1. Introduction

Primary liver cancer is the sixth most prevalent cancer and the second leading cause of cancer mortality worldwide [1]. The most common primary liver cancer is hepatocellular carcinoma (HCC), which accounts for >85% of all cases [2]. HCC predominantly arises in the setting of cirrhosis associated with hepatitis B and C virus infections, alcohol abuse, non-alcoholic steatohepatitis and metabolic diseases [3,4]. Early-stage HCC patients are often subjected to surgical resection and liver transplantation. However, 5-year recurrence rates after resection reach >70% [5] and the efficiency of transplantation is limited by organ shortage and technical issues [6]. Although local ablation and transarterial chemoembolization respectively confer 5-year survival rates of 50–70% and survival benefit of >6 months, they are confined to patients with single tumors or multinodular tumors with good liver reserve [7,8]. Chemotherapy is not routinely used due to the chemoresistant character of HCC [9]. For advanced HCC, sorafenib, a multi-tyrosine kinase inhibitor (MTKI), has been the only approved systemic first-line agent for over a decade, until lenvatinib, another MTKI, recently came into play. Yet, their clinical efficacy was suboptimal, with only ~3 months of prolonged survival [10,11]. Second-line treatments include regorafenib and cabozantinib, also MTKIs [12,13]; nivolumab and pembrolizumab, immune checkpoint inhibitors targeting programmed death-1 (PD-1) [14,15]; and ramucirumab, the first biomarker-driven therapeutics approved for HCC that targets the angiogenic vascular endothelial growth factor receptor (VEGFR) [16], all of which have not been established until the past few years and require further investigations. So far, treatment outcomes for HCC are far from satisfactory, with a 5-year survival rate of only ~18% [17]. Evidently, there is an urgent need to develop more effective therapeutic strategies in HCC.

Signal transducer and activator of transcription 3 (STAT3) has recently emerged as a potential therapeutic target for HCC due to its crucial roles in oncogenesis. STAT3 was initially determined to control acute-phase genes in response to interleukin-6 (IL-6) and epidermal growth factor (EGF) during inflammation [18]. It belongs to the STAT family of cytoplasmic transcription factors that mediate signal transduction from the plasma membrane to the nucleus in various cellular activities [19]. The STAT family comprises seven members: STAT1, 2, 3, 4, 5a, 5b and 6. Each of them consists of (i) an N-terminal domain for oligomerization, (ii) a coiled-coil domain for interaction with regulatory proteins, (iii) a DNA-binding domain for recognition of specific DNA sequences, (iv) a Src homology-2 (SH2) domain that triggers phosphorylation and dimerization after docking to phosphorylated receptors and (iv) a C-terminal transactivation domain with specific tyrosine (Y) (present in all STATs) and serine (S) residues (absent in STAT2 and 6) that are phosphorylated upon transcriptional activation [19,20]. 

Intensive investigation has been done on STAT3 since its discovery, revealing its physiological roles in early embryonic development, growth and differentiation of various adult tissues [21]. In addition, its pathogenic roles in cancer initiation, progression, metastasis, chemoresistance and immunoevasion have been uncovered [22]. To date, STAT3 is widely recognized as an oncogenic factor in diverse human cancers. Therefore, targeting STAT3 might be an attractive therapeutic strategy for HCC treatment. In this review, we summarize the oncogenic roles of STAT3 in HCC and the current clinical development of STAT3-targeted therapeutics.

## 2. The STAT3 Signaling Pathway

### 2.1. Activation and Regulation of STAT3

**Figure 1:**
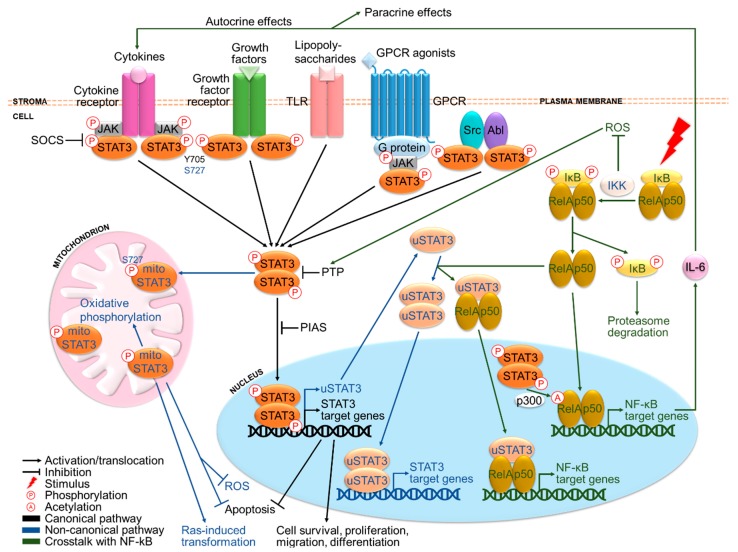
The STAT3 signaling pathway and its crosstalk with NF-kB. STAT3 is activated primarily by cytokines and growth factors, in addition to other signaling molecules. Canonically, ligand binding to receptors triggers phosphorylation of tyrosine kinases and subsequently STAT3 at Y705, followed by STAT3 dimerization and translocation to the nucleus where it drives transcription of target genes involved in cell survival and proliferation. Non-canonically, STAT3 can also be phosphorylated at S727, translocate to the mitochondrion, as well as autoregulate its own transcription to produce u-STAT3. Under normal conditions, STAT3 activation is under tight negative regulation by SOCS, PTP and PIAS members. Remarkably, STAT3 is involved in extensive crosstalk with the inflammatory NF-kB pathway. Activated NF-kB has been reported to either activate or inhibit STAT3 signaling, respectively by producing various cytokines including the major STAT3-inducing cytokine IL-6 and preventing reactive oxygen species (ROS) accumulation responsible for oxidizing negative regulators of STAT3. In return, STAT3 may sustain NF-kB activation via p300-mediated acetylation. Moreover, u-STAT3 and u-NF-kB can work in concert to coregulate another set of genes.

Essentially, STAT3 is a transcription factor that activates survival and proliferation signaling upon cytokine and growth factor stimuli. STAT3 can signal through both canonical and non-canonical pathways (Figure 1). Canonically, the binding of ligands to their cognate receptors leads to the recruitment and phosphorylation of tyrosine kinases, which recruit and phosphorylate STAT3 at Y705 (pY705) [21]. Subsequently, STAT3 proteins dimerize and translocate to the nucleus where they bind to promoter elements of target genes and modulate their transcription [21]. These include cell cycle regulatory genes such as *fos*, *cyclin D*, *c-Myc*, *pim1* and anti-apoptotic genes such as *B-cell CLL/Lymphoma-2* (*Bcl-2*), *Bcl-xL*, *survivin* and *X-linked inhibitor of apoptosis protein* (*XIAP*) [23]. Non-canonically, STAT3 may function independently of pY705 and nuclear localization (Figure 1). pS727 is required for maximal activation although pY705 plays a key activating role [24,25]. pS727 can also stimulate mitochondrial STAT3, where it may trigger oxidative phosphorylation [26], confer stress protection by reducing reactive oxygen species (ROS) accumulation and apoptosis [27,28] and support Ras-induced malignant transformation [29]. STAT3 can also autoregulate its own transcription, which will be discussed further in the next section.

STAT3 is primarily activated by ligand binding to (i) cytokine receptors and (ii) growth factor receptors, (iii) toll-like receptors (TLRs) [30], (iv) G-protein coupled receptors (GPCRs) [31] and (v) cytoplasmic tyrosine kinases such as Src and Abl [32,33] (Figure 1). A notable example is the proinflammatory cytokine IL-6, which binds to IL-6R/gp130 receptors and stimulates Janus kinases (JAKs), leading to STAT3 activation [34]. Other key STAT3-activating molecules include IL-6 family cytokines like IL-22 [35] and growth factors including EGF and vascular endothelial growth factor (VEGF) [36,37]. Except for growth factor receptors with intrinsic receptor tyrosine kinases (RTKs) and cytoplasmic kinases, other receptors rely on JAKs for phosphorylation. Under normal conditions, STAT3 activation is strictly governed by negative regulators, namely suppressor of cytokine signaling (SOCS) proteins [38], protein inhibitor of activated STAT (PIAS) proteins [39] and protein tyrosine phosphatases (PTPs) such as Src-homology-2 protein phosphatase-1 and 2 (SHP1/2) [40].

### 2.2. Crosstalk between STAT3 and NF-κB

Particularly, activation of STAT3 has been found to be associated with nuclear factor-kappa B (NF-kB), a family of inflammatory transcription factors consisting of NF-κB1 (p50), NF-κB2 (p52), Rel-A (p65), Rel-B and c-Rel subunits [41]. In response to proinflammatory stimuli, NF-kB translocates from the cytoplasm to the nucleus after being released from inhibitor of NF-κB (IκB), which is then phosphorylated by IκB kinase (IKK) and degraded (Figure 1). Activated NF-kB increases secretion of a panel of cytokines including IL-6 at the sites of inflammation [34], which activates STAT3 signaling. In fact, the NF-kB/IL-6/STAT3 pathway plays an important oncogenic role in cancers that arise from chronic inflammation such as HCC [42]. By contrast, NF-κB activation has also been shown to inhibit STAT3 signaling by preventing ROS accumulation responsible for oxidizing PTPs [43,44,45]. On the other hand, activated STAT3 can directly bind to the major NF-κB subunit Rel-A and promote its p300-dependent acetylation, causing prolonged nuclear localization and constitutive activation of NF-κB [34]. Moreover, STAT3 autoregulation produces unphosphorylated STAT3 (u-STAT3) molecules that bind to and facilitate nuclear translocation of unphosphorylated NF-κB (u-NF-κB), coregulating another set of target genes such as *IL-6*, *IL-8*, mesenchymal-epithelial transition factor (*MET*) and muscle RAS (*MRAS*) [46].

## 3. The Roles of STAT3 in HCC

### 3.1. Hepatic STAT3 Functions

Under physiological conditions, STAT3 is only transiently activated under the tight control of negative regulators and exerts various functions in the liver. In hepatocytes, the IL-6/STAT3 pathway is involved in hepatoprotection upon liver damage [35,47] and glucose homeostasis by inhibiting gluconeogenesis upon increase in plasma insulin [48,49]. In non-parenchymal hepatic cells, STAT3 activation by different stimuli also offers protective effects against cell injury, including cholangiocytes [50], stellate cells [51], endothelial cells [52] and liver-specific immune cells [53,54].

### 3.2. Clinical Implication of STAT3 in HCC

In contrast to transient activation in physiological states, STAT3 becomes persistently activated in the majority of malignancies [55]. Importantly, overexpression and constitutive activation of STAT3 has been found to be closely associated with pathogenesis and survival outcomes of HCC. He et al. has reported that ~60% of HCC cases exhibit nuclear STAT3 pY705 in tumoral but not surrounding non-tumoral tissues [44]. In addition, overexpression of STAT3 pY705 and/or pS727 in tumoral tissues has been found to be correlated with poor prognosis and clinicopathological features including larger tumor size, vascular invasion, advanced disease stage and cirrhosis in HCC patients [56,57,58,59]. Moreover, a significant association has been reported between STAT3 activity in stromal monocytes and poor prognosis in HCC, indicating the role of STAT3 in regulating the tumor microenvironment [60]. These data clearly substantiate the clinical significance of STAT3 in HCC.

Constitutive STAT3 activation in HCC could be explained by several causes. The foremost reason would be the elevated levels of STAT3-inducing signals, particularly, IL-6 and IL-22, which exert oncogenic functions via STAT3 activation in HCC [57,61,62]. Second, disruption of negative regulators of STAT3, such as SOCS3 and SHP1/2, may enhance STAT3 activation and promote HCC development [44,63]. Third, activating mutations in the gene encoding the gp130 subunit of IL-6R in benign hepatic adenomas have been found to cause STAT3 activation and HCC development when accompanied by β-catenin mutations, albeit at low frequency [64]. Intriguingly, no oncogenic mutations of STAT3 or JAKs have yet been detected in HCC [64].

### 3.3. Functions of STAT3 in HCC

**Figure 2:**
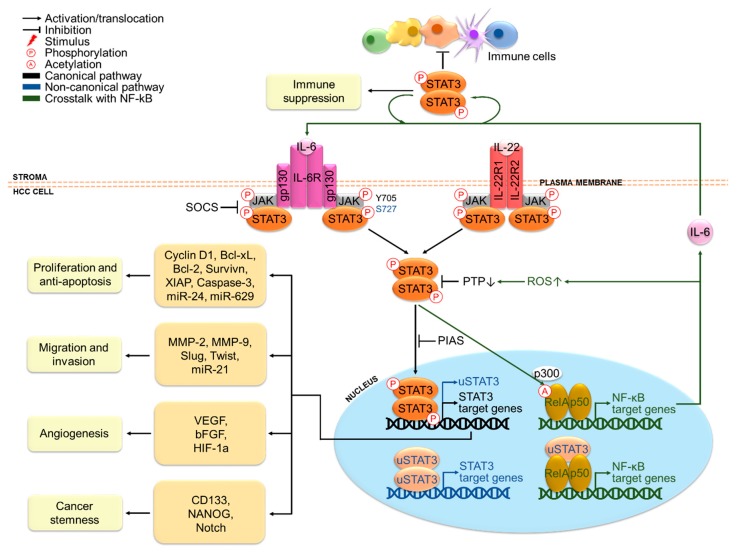
STAT3-mediated oncogenesis in hepatocellular carcinoma (HCC). IL-6 and IL-22 are major stimuli that activate STAT3 signaling in HCC cells. Activated STAT3 promotes HCC cell proliferation, anti-apoptosis, migration, invasion, angiogenesis and stemness properties via transcriptional regulation of target genes, cooperation with NF-kB and epigenetic regulation involving miRNA. A paracrine STAT3 activation loop between tumor and stromal cells also exerts suppressive effects in various immune cells in the HCC tumor microenvironment.

Indeed, the oncogenic functions of STAT3 in HCC have been extensively reported with relation to cancer cell proliferation, anti-apoptosis, migration, invasion, angiogenesis, stemness properties and immune suppression (Figure 2). These functions are mainly exerted via transcriptional regulation of different oncogenic target genes. The cooperation between STAT3 and NF-kB may also occur, given that around one third of HCC tumors display concomitant activation of STAT3 and NF-kB [44]. In addition, STAT3-mediated microRNA (miRNA) expression is also emerging as an epigenetic mechanism for driving hepatic oncogenesis and in turn, miRNA can also play a role in the regulation of STAT3 signaling [65].

In terms of proliferation and anti-apoptosis, STAT3 antisense oligonucleotides have been reported to inhibit proliferation and survival of several HCC cell lines [66]. In the same study, they have also been shown to impede tumorigenicity of a highly tumorigenic HCC cell line upon transplantation into mice [66]. Likewise, diethylnitrosamine (DEN)-induced HCC cells transduced with STAT3 short hairpin RNA (shRNA) have failed to form subcutaneous HCC tumors when transplanted into mice [44]. Besides, hepatocyte-specific STAT3-deficient mice have shown reduced size and multiplicity upon DEN treatment [44]. These results strongly justify the importance of STAT3 in HCC growth and tumor formation. Moreover, an epigenetic circuit involving multiple miRNAs has been demonstrated to promote HCC formation [67]. A key event in this mechanism is the downregulation of the hepatocyte nuclear factor 4α (HNF4α), a suppressor of hepatic oncogenesis, via IL-6/STAT3-dependent activation of miR-24 and miR-629. In turn, HNF4α-regulated expression of miR-124 is switched off, which releases its negative regulation on IL-6R, forming a positive feedback loop. In fact, the pro-proliferative role of STAT3 is highly related to its anti-apoptotic functions on HCC cells. Abrogation of STAT3 signaling by the Jak2 inhibitor, AG490, has triggered cell cycle arrest at G0/G1 phase in HCC cells via cyclin D1 downregulation and induced apoptosis by downregulating anti-apoptotic proteins Bcl-xL, survivin and XIAP [68]. STAT3 antisense treatment of HCCLM3 cells has also markedly impaired STAT3-dependent transcription of these genes and activated the main apoptosis executor caspase-3, leading to induction of apoptosis [66]. These studies provide support for the role of STAT3 in driving cell cycle progression and blocking apoptosis of HCC cells. By contrast, in mice transplanted with a highly metastatic HCC cell line and IL-22^+^ tumor-infiltrating lymphocytes from HCC patients, increased expression of pSTAT3 in tumor tissues has been detected along with an upregulation of cyclin D1, Bcl-2 and Bcl-xL, indicating the role of IL-22-mediated STAT3 activation in HCC tumor growth and resistance to apoptosis [57]. Collectively, these findings underpin the significance of STAT3 in HCC initiation and development. 

In terms of migration and invasion, it has been shown that STAT3 antisense treatment has reduced the invasiveness of HCC cells via downregulation of matrix metalloproteinases (MMP)-2 and MMP-9, which are engaged in the digestion of extracellular matrices [66]. Conversely, STAT3 activation has enhanced migration and invasion of HCC cells by transcriptionally inducing epithelial-to-mesenchymal transition (EMT) markers including Slug and Twist [69,70], suggesting that STAT3 activation may induce invasion and metastasis via mediation of EMT in HCC. In addition, the role STAT3 in migration and invasion has also been found to involve regulation of miR-21 and its targets [71].

In terms of angiogenesis, mice bearing STAT3 antisense-transfected HCC tumors have displayed decreased microvessel density and reduced circulating proteins of VEGF and basic fibroblast growth factor (bFGF), which are potent inducers of angiogenesis [66]. This clearly suggests the pro-angiogenic role of STAT3 in HCC. Mechanistically, STAT3 upregulates and recruits hypoxia inducible factor (HIF)-1α to form a transcriptional complex that binds to the VEGF promoter, thus inducing VEGF expression under hypoxia [72]. Indeed, increased VEGF expression has been correlated with poor prognosis in HCC patients, which is in accord with the marked vascularity characteristic of advanced HCC [73].

In terms of cancer stemness, IL6/STAT3 signaling has been found to induce expression of the cancer stem cell (CSC) marker CD133 via interaction of STAT3 with NF-κB and HIF-1α in HCC [74]. Besides, CD24-mediated STAT3 activation has been reported to regulate expression of the stem cell-associated protein, NANOG [75]. STAT3 can also promote stemness in HCC cells by activating Notch signaling, which is implicated in self-renewal and proliferation of CSCs [76]. These results reveal an important role of STAT3 in maintaining HCC stem cell phenotypes, which confer chemoresistance and contribute to recurrence upon chemotherapy treatment.

In terms of immune suppression, signaling molecules produced in the tumor microenvironment may establish a reciprocal STAT3 activation loop between tumor and stromal cells in a paracrine fashion, which exerts suppressive effects in various immune cells in HCC. STAT3 activation promotes various protumorigenic effects in stromal cells in HCC, including the production of immunosuppressive molecules from dendritic cells [77], polarization of tumorigenic M2 macrophages to antitumorigenic M1 subtype [78], cancer-associated fibroblast-mediated generation of myeloid-derived suppressor cells, inhibition of T cell proliferation and functions [79] and impairment of natural killer cell-mediated cytotoxicity [80]. These contribute to impaired effectiveness of immune surveillance against HCC.

Interestingly, studies have also revealed antioncogenic roles of STAT3 in RAS-dependent HCC [81] and early-stage HCC developed from carbon tetrachloride (CCl_4_)-induced liver fibrosis [82], implying that specific genetic context and etiology of the disease may impact the outcome of STAT3-targeted therapeutics. Nonetheless, as appreciated from the abundance of evidence illustrating the oncogenic roles of STAT3 in HCC, STAT3 is recognized as a vital oncogene in HCC and may serve as a potential therapeutic target for HCC therapy.

## 4. Clinical Trials of STAT3 Targeting Therapies

Given the constitutive activation and critical oncogenic roles of STAT3 in HCC and other cancer types, the STAT3 signaling pathway has emerged as a promising target for pharmacological intervention in cancer treatment. A myriad of STAT3-targeted drugs has been developed, which can be categorized into six major classes: (i) N-terminal domain inhibitors, (ii) DNA-binding domain inhibitors, (iii) SH2 domain inhibitors, (iv) antisense molecules, (v) inhibitors of downstream target genes and (vi) inhibitors of upstream activators or regulators. The first five classes are direct STAT3 inhibitors which respectively prevent interaction with regulatory proteins, DNA binding ability, phosphorylation and dimerization, protein expression and gene transcription. The sixth class acts indirectly by inhibiting upstream receptors such as JAK1/2 or stimulating negative regulators such as SHP1/2, of which a detailed analysis would be beyond the scope of this review due to its relative non-specificity for STAT3. Indeed, many of them have been demonstrated to exert antioncogenic effects in preclinical models of HCC and other cancers [83,84]. The remainder of this review will focus on direct STAT3 inhibitors that have advanced into clinical trials with promising therapeutic potential as monotherapy or in combination with other treatment modalities in HCC and other cancers.

### 4.1. Napabucasin: Cancer Stemness Inhibitor Targeting STAT3-Driven Gene Transcription

Napabucasin (BBI608) is the most extensively investigated STAT3-targeted agent against cancers thus far. It is a first-in-class cancer stemness inhibitor that inhibits transcription of STAT3 downstream target genes. In preclinical settings, it has shown to reduce the expression of stemness genes *β-catenin*, *NANOG*, *smoothened* and *sex-determining region Y-box protein 2* (*Sox2*), impede self-renewal and survival of various cancer cells including HCC cells in vitro, as well as prevent cancer relapse and metastasis in vivo [85]. Besides, it has also been demonstrated to sensitize stemness-high gastric cancer cells to standard chemotherapeutic agent paclitaxel [86]. 

Napabucasin is the only agent that has advanced into phase III trials among other STAT3-targeted therapeutics (Table 1). This orally administered drug has been proven safe at 240–480/500 mg twice daily (*n* = 41) (NCT01775423) [87] and has shown promising antitumor effects and potential to sensitize patients to conventional therapies in various cancers. Unfortunately, no studies have yet been reported on its clinical impact in HCC patients. The antitumor efficacy of napabucasin as a monotherapy has been particularly observed in colorectal cancer (CRC) among others. It has demonstrated a disease control rate (DCR) of 67% in CRC patients and 29% stable disease (SD) in other solid cancer patients in a phase Ib study (*n* = 24) (NCT01775423) [88]. It has also resulted in improved overall survival (OS) of advanced refractory CRC patients from 3 to 5.1 months after pSTAT3 stratification in another phase III trial (*n* = 46) (NCT01830621) [89].

In combination with chemotherapeutic drugs, full-dose napabucasin (500 mg, twice daily) and paclitaxel has passed safety and tolerability tests and shown encouraging clinical responses in patients with different advanced solid tumors, including gastric and gastroesophageal junction (GEJ) adenocarcinoma [91], platinum-resistant ovarian cancer (PROC) [92], pancreatic ductal adenocarcinoma (PDAC) [93], triple-negative breast cancer (TNBC) [94] and other cancer types [90] in individual cohorts of a phase Ib/II trial (NCT01325441). Particularly, in the gastric and GEJ adenocarcinoma cohort, this combination has shown promising results in both taxane-naïve (*n* = 16) and taxane-exposed patients (*n* = 19), with DCR being 75% versus 68% [91]. When combined with gemcitabine plus nab-paclitaxel, half-dose napabucasin (240 mg, twice daily) has been proven to be safe, with 93% DCR, 80% tumor regression and 47% partial response (PR) in metastatic PDAC patients in a phase Ib/II trial (*n* = 37) (NCT02231723) [99]. The combination of folinic acid-5-fluorouracil-irinotecan (FOLFIRI) regimen in the presence or absence of bevacizumab with half-dose napabucasin has also been well tolerated in advanced CRC patients. In a phase Ib/II study (NCT02024607) [97], the DCR of FOLFIRI-naïve (*n* = 34) and FOLFIRI-exposed patients (*n* = 29) has been 82% versus 72%. Interestingly, patients with pSTAT3^high^ (*n* = 30) and pSTAT3^low^ (*n* = 27) statuses have shown DCR of 83% and 89% respectively, suggesting possible synergism between napabucasin and FOLFIRI irrespective of pSTAT3 status. The result of its extension study has also been positive, with DCR of 90% in FOLFIRI-exposed patients (*n* = 19) [98].

The targeted drug panitumumab, a human anti-EGFR monoclonal antibody, could also be safely combined with full-dose napabucasin in KRAS wild-type metastatic (m) CRC as reported in a phase Ib/II trial (NCT10776307) [95,96]. This combination has shown positive antitumor activity in both anti-EGFR-naïve patients (*n* = 24) and those who failed anti-EGFR therapy (*n* = 48), with DCR being 48% versus 59% and PFS being 16.9 weeks versus 9 weeks respectively, suggesting that prior anti-EGFR exposure is not limiting the efficacy of this combination therapy and that napabucasin may sensitize patients to repeat anti-EGFR therapy.

Apart from good antitumor activity, napabucasin has shown a manageable toxicity profile in patients with various cancers. The most common adverse events have been mild gastrointestinal symptoms, such as diarrhea, nausea and vomiting, which could be generally kept under control by antidiarrheal and antiemetic agents. At present, different trials are ongoing to assess the efficacy of combination regimens of napabucasin with (i) chemotherapeutic drugs, including paclitaxel, gemcitabine plus nab-paclitaxel and FOLIFIRI, (ii) the standard systemic targeted drug for advanced HCC, sorafenib and (iii) immune checkpoint inhibitors ipilimumab, nivolumab and pembrolizumab. Besides, the pro-drug of napabucasin, DSP-0337, is now being evaluated for its safety, tolerability, pharmacokinetics and antitumor activity in a phase I trial for advanced solid tumors (NCT03416816) and may serve as an alternative for napabucasin. Although clinical benefits of napabucasin has been demonstrated in different types of solid tumors, an important unmet need exists to gain better understanding of its clinical effects in HCC patients.

### 4.2. AZD9150: STAT3-Targeted Antisense Oligonucleotide

AZD9150 (ISIS481464), a STAT3-targeted antisense oligonucleotide that reduces STAT3 mRNA expression, has also shown promising antioncogenic effects. Preclinical findings have demonstrated its ability to decrease the expression of *STAT3* and its downstream oncogenic target genes in a broad range of cancer cells [100]. In particular, this drug has been effective against leukemia and lymphoma both in vitro and in vivo [100,101], while it has also inhibited primary or secondary tumor growth respectively in xenografts of lung cancer and neuroblastoma [100,102].

Clinically, the only study of AZD9150 in HCC completed thus far is a phase I trial evaluating its safety and antitumor activity in patients with advanced or metastatic HCC (*n* = 58) (NCT01839604) (Table 2) [103]. It has been well tolerated at doses up to 3 mg/kg, applying 3 infusions in the first week followed by weekly infusions, with only mild and few serious adverse reactions. Of note, another phase I trial in treatment-refractory cancer patients, with half of them suffering from advanced lymphoma and the others having various types of solid tumors, has reported promising therapeutic effects (*n* = 25) (NCT number not stated) [100]. The DCR has reached 44%, including 3 patients with diffuse large B-cell lymphoma (DLBCL) displaying tumor shrinkage and 2 patients showing durable PR. Besides, 33% of the patients have displayed >30% of post-treatment reductions in circulating concentrations of IL-6. IL-6 is a prime stimulus for STAT3 activation and elevated serum levels of IL-6 have been associated with poor prognosis in various cancers [104]. Thus, such decline in circulating IL-6 may serve as an indicator of STAT3 pathway inhibition. Encouraging results have also been described in a more recent phase I trial in relapsed or refractory lymphoma patients primarily consisting of a DLBCL population (*n* = 30) (NCT01563302) [105]. The DCR has been 17%, including 2 complete responses with median response duration of 10.7 months, 2 PRs and 1 SD.

In general, AZD9150 is deemed safe and effective in advanced cancer patients especially those with lymphoma. There have been few unacceptable toxicity events, with the most common adverse events being elevated levels of aspartate and alanine aminotransferase and thrombocytopenia. Many phase I/II trials of AZD9150 are now underway in numerous cancer types including solid tumors and hematological malignancies, either as a single agent or in combination with chemotherapeutic, targeted or immunomodulatory agents. Nonetheless, like napabucasin, clinical investigations of AZD9150 in HCC are still in its infancy and further efforts are urgently needed.

### 4.3. OPB Compounds, Pyrimethamine and TTI-101: STAT3 SH2 Domain Inhibitor

In the realm of SH2 domain inhibitors, OPB-31121 is one of the earliest oral drugs developed by Otsuka Pharmaceuticals. It prevents STAT3 dimerization upon phosphorylation by binding with high affinity to the SH2 domain of STAT3 [106]. Preclinically, OPB-31121 has demonstrated good antitumor activity in leukemic [107] and gastric cancer cells [108] and even elicited synergistic effects against gastric cancer when combined with chemotherapeutic agents [108]. However, a phase I clinical trial in patients with advanced solid tumors has not yielded any objective responses, accompanied with a highly varied pharmacokinetic profile (*n* = 14) (NCT00955812) [109] (Table 3). As such, further development of the compound has been discontinued.

In the same phase I study, OPB-111077, the primary metabolite of OPB-31121, has been found to be accumulated at higher tissue levels [109]. In vitro studies have shown that OPB-111077 profoundly inhibits the growth of various cancer cell types [109]. Subsequently, two phase I trials have been conducted in patients with advanced HCC and other solid tumors respectively (Table 3). Although no objective responses have been achieved in the HCC trial (*n* = 33) (NCT01942083) [111], antitumor responses have been more encouraging in the other study, with one PR in a DLBCL patient and 39% patients showing SD or minor responses (*n* = 145) (NCT01711034) [110]. In both studies, the drug has been well-tolerated and the recommended phase II dose has been determined to be 250 mg once daily. These indicate the feasibility of STAT3 inhibition with OPB-111077 but more trials are essential for assessing its safety, pharmacokinetics and therapeutic efficacy in larger cohorts of patients with HCC or other cancer types. Results of two recently completed studies in advanced solid tumors (NCT02250170) and relapsed/refractory acute myeloid leukemia (AML) (NCT03197714) are eagerly awaited. Three other ongoing studies are evaluating OPB-111077 either as a single agent or in combination with chemo- and targeted therapy.

On the other hand, OPB-51602 has also demonstrated inhibitory effects against different cancer models [115] but intolerability issues of this drug have been revealed in the clinic (Table 3). A phase I study in refractory solid tumors has demonstrated poorer tolerability for continuous dosing, compared with intermittent dosing at 4 mg daily with two weeks of treatment followed by one week of rest (*n* = 51) (NCT01184807) [112]. Modest antitumor responses have been reported, with two patients showing PR at 5 mg intermittently and 4 mg continuously, both of whom suffer from EGFR mutation-positive non-small-cell lung cancer with prior EGFR-TKI therapy. The recommended phase II dose has also been determined to be 4 mg in another study in relapsed/refractory hematological malignancies (*n* = 20) (NCT02058017) [113]. However, because of poor tolerability and lack of responses in long-term daily administration at higher doses, further clinical development of the drug with daily dosing in hematological malignancies has been halted. Another study in advanced nasopharyngeal carcinoma (NPC) has also been terminated due to patient intolerability to lactic and metabolic acidosis elicited by the drug (*n* = 9) (NCT02058017) [114]. There has not been published record for two other completed phase I trials, including a safety and tolerability study in advanced cancers (NCT01423903) and a pharmacodynamic and pharmacogenetic biomarker study in advanced solid tumors (NCT01867073).

While OPB-111077 is probably the most promising OPB candidate by far, pyrimethamine and TTI-101 are two other STAT3 SH2 domain inhibitors currently undergoing early phase clinical trials in relapsed chronic lymphocytic leukemia (CLL) or small lymphocytic leukemia (SLL) (NCT01066663) and other advanced cancers including HCC (NCT03195699) respectively (Table 3). Both of them have shown significant inhibitory effects in preclinical breast cancer models [116,117].

### 4.4. STAT3 Decoy: STAT3-DNA Binding Inhibitor

STAT3 decoy is a 15-bp double-stranded oligonucleotide that competitively inhibits STAT3 binding to the response element within the c-fos promoter. It has been shown to effectively induce apoptosis, suppress growth and downregulate STAT3 target genes in in vitro and in vivo cancer models, including liver [118], lung [119,120], head and neck [121,122] and ovarian cancer [123]. Notably, Sen et al. has conducted the first phase 0 clinical trial in head and neck tumors from patients undergoing surgical resection (*n* = 32) and reported that intratumoral injection of STAT3 decoy abrogates expression of STAT3 target genes including *cyclin D1* and *Bcl-xL* without toxicities [122]. Although preliminary, this study may serve as a basis for future endeavors in the application of STAT3 decoy in biopsies of HCC or other solid tumors and in more advanced clinical settings.

### 4.5. Current Status and Future Perspectives of STAT3-Targeted Therapies in HCC

The existing preclinical and clinical evidence strongly justifies the use of STAT3-targeted drugs as a promising therapeutic approach against HCC. To date, napabucasin is considered the most potent STAT3 inhibitor with an acceptable safety profile in the clinic. Although the STAT3-targeted antisense oligonucleotide, AZD91150, have demonstrated antitumor activities, its efficient delivery and stability in vivo remain technically challenging. For SH2 domain inhibitors, the development of OPB compounds is somewhat limited by tolerability issues, whereas others are still under early clinical investigation. Proof of concept for DNA-binding domain inhibition by STAT3 decoy has sufficed to warrant further clinical studies but it shares the same concerns as antisense oligonucleotides. Despite these limitations, direct STAT3 inhibitors are better options than indirect inhibitors targeting upstream regulators of STAT3, as the latter lack specificity to the STAT3 pathway and may produce a range of undesired pleiotropic effects. While many direct STAT3 inhibitors have not been clinically well explored in HCC, current clinical data in various other cancers suggest their potential benefit in HCC patients.

Importantly, a combinational approach for STAT3-targeted agents may be more effective than STAT3-targeted monotherapy. It has been elucidated that STAT3 is involved in extensive crosstalk with other signaling pathways and that single activating mutation of STAT3 is rare in HCC. Combination of STAT3-targeted drugs with other anticancer therapeutics may address these issues by simultaneously targeting different mechanisms of action, thus eliciting more powerful antitumor responses. While targeted agents specifically block molecular pathways that promote oncogenesis, chemotherapeutic and immunotherapeutic agents respectively inhibit growth of all dividing cells and stimulate immune responses to attack tumor cells. So far, napabucasin is the only STAT3-targeted drug that has been clinically studied in combination with other therapies. As discussed before, early-phase trials have revealed promising antitumor efficacy when combining napabucasin with standard chemodrugs including paclitaxel [90,91,92,93,94], gemcitabine plus nab-paclitaxel [99] and FOLFIRI with or without bevacizumab [97,98], as well as another targeted drug panitumumab [95,96] in several solid malignancies. Preclinical results have also suggested that napabucasin may synergize with paclitaxel to overcome drug resistance [86] and sensitize CRC to immune checkpoint inhibitors in syngeneic tumor models [124]. These findings support the notion that napabucasin may sensitize refractory cancer patients to chemotherapy, other targeted therapy and immunotherapy. Given that therapeutic resistance is a common phenomenon in advanced HCC, a combinational approach for STAT3-targeted drugs may be more effective than monotherapy. Notably, blockade of the immune checkpoint, programmed death receptor-1 (PD-1), with the fully human monoclonal antibody nivolumab has been shown to safely induce durable objective responses in patients with cancer types including HCC [14,125]. Thus, strategies to combine STAT3-targeted drugs with immune checkpoint inhibitors, which reactivate immune responses from suppression, may be of great value. Nevertheless, it must be emphasized that current clinical studies of STAT3-targeted agents have been chiefly based on cancer types other than HCC and more efforts to evaluate their clinical performance in HCC are strongly urged.

Apart from combinational strategies, novel STAT3 inhibitors and better biomarker strategies may improve the therapeutic efficacy of STAT3-targeted agents. It is expected that advancement in technology, for instance, high-throughput screening platforms of protein-protein interaction inhibitors, would boost the discovery of novel STAT3-targeted drugs. As STAT3 is a pivotal regulator of cellular metabolism under physiological conditions, an ideal STAT3-targeted agent should minimize its toxicities in normal cells while preserving its specificity and efficacy against tumor-associated components. Besides, a more robust biomarker strategy should be established for patient stratification. Although overexpression of STAT3 pY705 is the typical definition of STAT3 activation, it may not be broadly representative. Further investigations on biomarkers of STAT3 activation may be helpful in identifying cancer patients for STAT3-targeted therapies and thus improve clinical outcomes.

## 5. Conclusions

HCC is an extremely deadly tumor and the search for innovative treatment strategies is never ending. Overexpression and constitutive activation of STAT3 in HCC tumors has been found to associate with disease development and patient prognosis. The oncogenic functions of STAT3 have also been well established in numerous HCC models. These preclinical and clinical findings provide rationale for the use of STAT3 as a novel therapeutic target in HCC. Various types of STAT3 inhibitors have since been developed. Currently, clinical trials evaluating different STAT3-targeted strategies as monotherapy or combination therapy are ongoing. Although encouraging results have been shown in various cancer types, clinical trials on STAT3-targeted therapies in HCC are still limited. This stresses the need for more assessments of STAT3 inhibitors in HCC patients, especially in combination with other anticancer therapeutics. Future development of novel STAT3 inhibitors with lower toxicities and higher efficacy is anticipated. Besides, more efforts are required to delineate a comprehensive STAT3-associated biomarker profile, which helps define a subset of HCC patients that are more susceptible to STAT3 inhibition.

## Figures and Tables

**Table 1:** Clinical trials of napabucasin: cancer stemness inhibitor targeting STAT3-driven gene transcription.

NCT No.	Phase	Therapy	Cancer Types	Brief Description	Time	Ref
NCT 01775423	Ib	Monotherapy	Advanced solid cancers (*n* = 41)	Dose escalation achieved	2009–2019	[87]
	Ib		Advanced solid cancers (*n* = 24)	Safety and antitumor activity demonstrated in higher strength capsule		[88]
NCT 01830621	III	Monotherapy	Advanced CRC (*n* = 280)	Prolonged OS demonstrated after pSTAT3 stratification	2013–2016	[89]
NCT 03416816	I	Monotherapy	Advanced solid tumors (*n* = 90)	To evaluate the safety, tolerability, pharmacokinetic profile and antitumor activity of pro-drug of napabucasin, DSP-0337	2018–2020	/
NCT 01325441	Ib/II	Combination	Advanced solid cancers (*n* = 24)	Safety and antitumor activity demonstrated with paclitaxel, particularly in gastric and GEJ adenocarcinoma	2011–2020	[90]
	Ib/II		Advanced gastric and GEJ adenocarcinoma (*n* = 46)	Safety and antitumor activity demonstrated with paclitaxel, regardless of prior taxane exposure		[91]
	Ib/II		Advanced PROC (*n* = 98)	Safety and antitumor activity demonstrated with paclitaxel, including 3 completes responses		[92]
	Ib/II		Advanced PDAC (*n* = 41)	Safety and antitumor activity demonstrated with paclitaxel, particularly in taxane-naïve group		[93]
	Ib/II		Advanced TNBC (*n* = 35)	Safety and antitumor activity demonstrated with paclitaxel, particularly in taxane-exposed therapy		[94]
NCT 01776307	Ib/II	Combination	KRAS-wt mCRC (*n* = 24)	Safety and antitumor activity demonstrated with panitumumab, regardless of prior anti-EGFR exposure	2012–2019	[95]
	II		KRAS-wt mCRC (*n* = 72)			[96]
NCT 02024607	Ib/II	Combination	Advanced CRC (*n* = 63)	Safety and antitumor activity demonstrated with FOLFIRI ± bevacizumab	2014–2019	[97]
	III		Advanced CRC (*n* = 46)	Safety and antitumor activity demonstrated with FOLFIRI ± bevacizumab, regardless of prior FOLFIRI ± bevacizumab exposure and pSTAT3 status		[98]
NCT 02231723	III	Combination	mPDAC (*n* = 37)	Safety and antitumor activity demonstrated with gemcitabine and nab-paclitaxel	2014–2020	[99]
NCT 02178956	III	Combination	Advanced gastric and GEJ adenocarcinoma (*n* = 680)	To determine if napabucasin in combination with paclitaxel prolongs OS than paclitaxel alone	2014–2019	/
NCT 02279719	Ib/II	Combination	Advanced HCC (*n* = 99)	To evaluate the safety, tolerability, pharmacokinetic profile and antitumor activity in combination with sorafenib in comparison with sorafenib alone	2014–2019	/
NCT 02358395	I	Combination	Advanced HCC (*n* = 12)	To evaluate the safety, tolerability, pharmacokinetic profile and antitumor activity in combination with sorafenib	2015–2019	/
NCT 02467361	III	Combination	Advanced cancers (*n* = 104)	To evaluate the safety, tolerability, pharmacokinetic profile and antitumor activity in combination with immune checkpoint inhibitors	2015–2019	/
NCT 02753127	III	Combination	mCRC (*n* = 1253)	To determine if napabucasin in combination with FOLFIRI prolongs OS than FOLFIRI alone	2016–2020	/
NCT 02993731	III	Combination	mPDAC (*n* = 1134)	To determine if napabucasin in combination with nab-paclitaxel and gemcitabine prolongs OS than nab-paclitaxel and gemcitabine alone	2016–2020	/
NCT 03416816	I	Monotherapy	Advanced solid tumors (*n* = 90)	To evaluate the safety, tolerability, pharmacokinetic profile and antitumor activity	2018–2020	/

**Table 2:** Clinical trials of AZD9150: STAT3-targetd antisense oligonucleotide.

NCT No.	Phase	Therapy	Cancer Types	Brief Description	Time	Ref.
NCT 01563302	I/II	Monotherapy	Predominantly refractory DLBCL (*n* = 30)	Safety and antitumor activity demonstrated	2012–2016	[105]
NCT 01839604	II	Monotherapy	Advanced HCC (*n* = 58)	Safety, tolerability and pharmacokinetics evaluated but with limited antitumor activity	2013–2015	[103]
Not stated	I	Monotherapy	Advanced lymphoma and solid tumors (*n* = 25)	Safety and antitumor activity demonstrated	2015	[100]
NCT 02499328	I & II	Combination	Advanced solid tumors (*n* = 465)	To evaluate the safety, tolerability and antitumor activity in combination with durvalumab, tremelimumab and AZD5069	2015–2020	/
NCT 02549651	1	Combination	Relapsed/refractory DLBCL (*n* = 32)	To evaluate the safety, tolerability and antitumor activity in combination with durvalumab and tremelimumab compared with durvalumab alone	2016–2019	/
NCT 02546661	I	Combination	MIBC (*n* = 156)	To evaluate the safety, tolerability and antitumor activity of durvalumab in combination with AZD9150 or other novel anticancer agents compared with durvalumab alone	2016–2020	/
NCT 02983578	II	Combination	Advanced pancreatic, lung, colorectal cancer (*n* = 75)	To evaluate the antitumor activity and tumor-based biomarkers in combination with durvalumab	2017–2021	/
NCT 03334617	II	Combination	Advanced non-small cell lung cancer (NSCLC) (*n* = 260)	To evaluate the safety, tolerability and antitumor activity of different combinations of anticancer agents	2017–2021	/
NCT 03394144	I	Combination	Advanced solid tumors (*n* = 110)	To evaluate the safety, tolerability and antitumor activity in combination with durvalumab compared with AZD9150 alone	2018–2019	/
NCT 03421353	I & II	Combination	Advanced solid tumors (*n* = 110)	To evaluate the safety, tolerability and antitumor activity in combination with durvalumab and chemodrugs compared with AZD9150 alone; to compare its bioavailability of subcutaneous and intravenous formulations	2018–2020	/
NCT 03742102	Ib/II	Combination	mTNBC (*n* = 110)	To evaluate the safety, tolerability and antitumor activity of durvalumab and paclitaxel in combination with AZD9150 or other novel anticancer agents	2018–2020	/
NCT 03819465	Ib	Combination	Advanced NSCLC (*n* = 200)	To evaluate the safety, tolerability and antitumor activity of durvalumab in combination with AZD9150 or other novel anticancer agents +/- chemotherapy	2018–2020	/
NCT 03527147	I	Combination	Relapsed/refractory non-Hodgkin lymphoma (*n* = 88)	To evaluate the safety, tolerability and antitumor activity of different combinations of targeted agents	2018–2021	/

**Table 3:** Clinical trials of OPB compounds, pyrimethamine, TTI-101: STAT3 SH2 domain inhibitors.

NCT No.	Phase	Therapy	Cancer Types	Brief Description	Time	Ref.
**OPB-31121**						
NCT 00955812	I	Monotherapy	Advanced solid tumors (*n* = 14)	Unfavorable pharmacokinetic profile and antitumor activity demonstrated, leading to discontinuation of compound development	2009–2012	[109]
**OPB-111077**						
NCT 01711034	I	Monotherapy	Advanced solid tumors (*n* = 145)	Safety, tolerability and antitumor activity demonstrated, including one PR in DLBCL	2012–2015	[110]
NCT 01942083	I	Monotherapy	Advanced HCC (*n* = 33)	Safety and tolerability demonstrated but no antitumor response was observed	2013–2017	[111]
NCT 02250170	I	Monotherapy	Advanced solid tumors (*n* = 47)	To evaluate the safety, tolerability and antitumor activity	2014–2019	/
NCT 03197714	Ib	Monotherapy	Relapsed/refractory AML (*n* = 15)	To evaluate the safety, tolerability and antitumor activity	2017–2018	/
NCT 03158324	IIa	Monotherapy	NPC/refractory tumors (*n* = 52)	To evaluate the safety, tolerability and antitumor activity	2017–2020	/
NCT 03063944	I	Combination	AML (*n* = 12)	To evaluate the safety, tolerability and antitumor activity in combination with decitabine	2017–2023	/
NCT 04049825	I	Combination	Relapsed/refractory DLBCL (*n* = 65)	To evaluate the safety, tolerability and antitumor activity in combination with bendamustine and rituximab	2019–2021	/
**OPB-51602**						
NCT 01184807	I	Monotherapy	Refractory solid tumors (*n* = 51)	Better tolerability for intermittent than continuous dosing; antitumor activity demonstrated, including 2 PRs in EGFR mutation-positive NSCLC prior anti-EGFR exposure	2009–2013	[112]
NCT 01423903	I	Monotherapy	Advanced cancers (*n* = 45)	To evaluate the safety, tolerability, pharmacokinetic profile and antitumor activity	2010–2013	/
NCT 01344876	I	Monotherapy	Relapsed/refractory hematological malignancies (*n* = 20)	Safety and tolerability demonstrated but long-term administration at higher doses was difficult with daily dosing and no antitumor response was observed, leading to termination of study	2011–2014	[113]
NCT 01867073	I	Monotherapy	Advanced solid tumors (*n* = 20)	To evaluate the pharmacokinetic profile in relation to pSTAT3 expression in peripheral mononuclear blood cells and single nucleotide polymorphisms in patient tissues	2011–2015	/
NCT 02058017	I	Monotherapy	Advanced NPC (*n* = 9)	Poor tolerability, leading to termination of study	2013–2015	[114]
**Pyrimethamine**						
NCT 01066663	I & II	Monotherapy	Relapsed CLL/SLL (*n* = 26)	To evaluate the safety, tolerability, pharmacokinetic profile and antitumor activity	2010–2024	/
**TTI-101**						
NCT 03195699	I	Monotherapy	Advanced cancers (*n* = 30)	To evaluate the safety, tolerability and pharmacokinetic profile	2017–2020	/
